# An Artificial Intelligence System for Optimizing Radioactive Iodine Therapy Dosimetry

**DOI:** 10.3390/jcm13010117

**Published:** 2023-12-25

**Authors:** Michalis F. Georgiou, Joshua A. Nielsen, Rommel Chiriboga, Russ A. Kuker

**Affiliations:** 1 Department of Radiology, University of Miami Miller School of Medicine, Miami, FL 33136, USA; 2 Department of Radiology, Jackson Memorial Hospital, Miami, FL 33136, USA; aapljosh@gmail.com (J.A.N.);; 3 Nuclear Medicine, Brooke Army Medical Center, San Antonio, TX 78234, USA

**Keywords:** thyroid cancer, iodine therapy, dosimetry, artificial intelligence

## Abstract

Thyroid cancer, specifically differentiated thyroid carcinoma (DTC), is one of the most prevalent endocrine malignancies worldwide. Radioactive iodine therapy (RAIT) using I-131 has been a standard-of-care approach for DTC due to its ability to ablate remnant thyroid disease following surgery, thus reducing the risk of recurrence. It is also used for the treatment of iodine-avid metastases. RAIT dosimetry can be employed to determine the optimal treatment dose of I-131 to effectively treat cancer cells while safeguarding against undesirable radiation effects such as bone marrow toxicity or radiation pneumonitis. Conventional dosimetry protocols for RAIT, however, are complex and time-consuming, involving multiple days of imaging and blood sampling. This study explores the use of Artificial Intelligence (AI) in simplifying and optimizing RAIT. A retrospective analysis was conducted on 83 adult patients with DTC who underwent RAIT dosimetry at our institution between 1996 and 2023. The conventional MIRD-based dosimetry protocol involved imaging and blood sampling at 4, 24, 48, 72, and 96 h post-administration of a tracer activity of I-131. An AI system based on a deep-learning neural network was developed to predict the maximum permissible activity (MPA) for RAIT using only the data obtained from the initial 4, 24, and 48 h time points. The AI system predicted the MPA values with high accuracy, showing no significant difference compared to the results obtained from conventional MIRD-based analysis utilizing a paired *t*-test (*p* = 0.351, 95% CI). The developed AI system offers the potential to streamline the dosimetry process, reducing the number of imaging and blood sampling sessions while also optimizing resource allocation. Additionally, the AI approach can uncover underlying relationships in data that were previously unknown. Our findings suggest that AI-based dosimetry may be a promising method for patient-specific treatment planning in differentiated thyroid carcinoma, representing a step towards applying precision medicine for thyroid cancer. Further validation and implementation studies are warranted to assess the clinical applicability of the AI system.

## 1. Introduction

Thyroid cancer remains the most prominent endocrine malignancy, with an increased number of incidences in the United States and all over the world [1,2,3]. Differentiated thyroid carcinoma (DTC) is the most common type [2,4], representing more than 95% of cases [5] and an estimated 30% of recurrences [4]. Differentiated thyroid cancer (DTC) originates from the follicular epithelium and maintains fundamental biological characteristics of normal, healthy thyroid tissue [6]. This property allows radioiodine pharmaceuticals to be used in both the diagnosis and treatment of DTC [6]. Molecular imaging based on planar gamma camera, SPECT, SPECT/CT, and PET/CT, can be performed with radioiodine isotopes such as I-123, I-131, and I-124 and can provide an accurate localization of sites with pathological uptake as in metastatic lesions or residual thyroid remnants in DTC following thyroidectomy [7]. Furthermore, radioiodine imaging and molecular imaging methods, in general, can provide visual representation, characterization, and quantification of the biological properties of patients’ cells and tissues [8] and can have significant clinical benefit in staging, risk stratification, and alteration of management of DTC [9]. The uptake mechanism of radioiodine is facilitated by thyroid cancer cells being able to efficiently trap circulatory iodine via the expression of sodium iodide symporter (NIS) [10]. In this regard, radioactive iodine treatment (RAIT) plays a key role in the overall management of DTC and has become the standard-of-care approach for the ablation of remnant thyroid disease following surgery to reduce the risk of recurrence [2] and for the treatment of iodine-avid metastases [11]. Commonly, there are two main approaches in determining the activity to be used for RAIT, each with its own advantages and limitations. The most commonly used approach is a fixed activity of I-131 between 1.1 and 3.7 GBq for the initial treatment of remnant ablation following thyroidectomy [12] or higher amounts up to 7.4 GBq for repeat treatments or for metastatic disease [13]. Researchers have also proposed thresholds in terms of whole-body retention at 48 h post-administration of a small diagnostic dose for increasing or decreasing the empirically derived activity so as not to exceed the maximum tolerated activity [14,15]. While an advantage of the fixed method for determining the dose is simplicity, the main limitation is that it does not take into consideration the individuality of the patient and that the prescribed dose may neither reflect the minimal activity that will deliver the effective dose treatment nor the maximum permissible and reasonably safe one [11]. An additional limitation of the empirically fixed-dose approach is that multiple treatments with fixed activities may not be as effective as a single treatment of the same total dose and also that multiple treatments may result in reduced effectiveness of subsequent treatments [16]. Contrary to the fixed activity method, the second approach utilizes dosimetry-based calculations to determine the treatment dose for each patient, considering individual factors related to the variability in the biokinetics of organs and lesions [5]. This approach can lead to personalized and more effective RAIT. The optimal treatment dose derived from dosimetry would administer the highest permissible and reasonably safe absorbed dose while also safeguarding against undesirable side effects [11,17]. Dorn et al. [18] used a dosimetry-guided RAIT approach in patients with DTC that enabled the administration of large safe doses based on a limit of 3 Gy to the red marrow and 30 Gy to the lungs. In the current era of radionuclide therapy and molecular theranostics, precision medicine and patient-specific treatment planning are gaining wide acceptance. Therefore, obtaining the optimal I-131 RAIT activity through dosimetry-based methods is particularly significant and can be crucial for successful patient treatment planning.

## 2. Dosimetric Approaches for RAIT

Presently, there are two dosimetric approaches to calculating the maximum permissible activity (MPA) for RAIT:

(a) Bone marrow dose limiting, originally introduced by Benua et al. in 1962 [19] and later by Leeper [20] and Benua and Leeper [21] and further improved with the introduction of the MIRD formulism (Medical Internal Radiation Dosimetry Committee of the Society of Nuclear Medicine). Bone marrow is the most radiosensitive tissue in the body and is commonly the dose-limiting one for radionuclide therapy [22]. The method is mainly concerned with the safety of the treatment and considers blood as a bone marrow surrogate [23]. This approach accepts a threshold of a blood-absorbed dose of 2 Gy (200 rad) to avoid myelotoxicity [24] and the generally accepted thresholds of a lesion-absorbed dose of 300 Gy for thyroid remnants and of 80 Gy for metastases. This method also takes into consideration the absorbed dose to the lungs, ensuring it remains below 30 Gy to avoid pulmonary fibrosis; this translates into a threshold of 4.4 GBq (120 mCi) as the whole-body retention at 48 h and of less than 3 GBq (80 mCi) for patients with iodine-avid diffuse lung metastases [21,24]. Based on this method, the activity in the whole body is measured using gamma camera imaging in conjugate views (anterior and posterior) following the administration of tracer activity. The frequency of the imaging scans and the time intervals for blood sampling are detailed in relevant publications [24] and usually involve imaging over a course of 4–5 days at time points of 2 h, 24 h, 48 h, 72 h, and 96 h post-administration, with some variations depending on the exact implementation. The activity in the blood is measured using serial blood sampling methods correlating with the imaging time points. Mathematical analysis is applied, usually through software, to integrate the various organ time–activity curves and to calculate the total absorbed dose of radiation to the blood [15]. The details of the formulism’s implementation are described in the MIRD pamphlets [25,26,27] and other relevant publications [11,22] and are beyond the scope of this manuscript. Software that implements the MIRD formulism was also developed [28].
(b) The lesion-based approach aims to improve the efficacy of treatment planning by delivering minimum absorbed doses of 300 Gy for remnant thyroid tissue and 80 Gy for metastases [29]. These thresholds were originally based on data presented by Maxon et al. [30] and Maxon and Smith [31]. The value of 80 Gy was originally defined for the treatment of cervical lymph node metastases and is assumed to be accurate for distant metastases. Using this method, dosimetric analysis can be performed post-treatment on thyroid remnant tissue or on lesions to assess the effectiveness of the treatment dose by correlating with clinical results [13]. An advantage of this method is its ability to ablate remnants with lower activities [29]. Uptake and clearance of I-131 from identifiable thyroid remnants and/or metastatic lesions can be generally measured using modeling that can be incorporated into software, such as the well-known OLINDA/EXM model [32].

## 3. Efforts to Optimize and Simplify Dosimetry Protocols

Most dosimetry protocols require whole-body imaging, blood sample measurements, and sometimes even urine collection over the course of 5 or more days to adequately assess the clearance or RAI in the patient. Dosimetry protocols are considered time-consuming and complex procedures with logistical complexities and inconvenience for the patient. These are some of the reasons that hinder the widespread adoption of dosimetry in RAIT, along with the requirement for expertise and proper dosimetric software tools. These limitations have motivated investigators to seek approaches to modify, simplify, and optimize dosimetric protocols. Hänscheid et al. [33] suggested dosimetry from a single time point of the external measurement of whole-body retention on day 1 or day 2 post-administration of radioiodine with the application of the MIRD formulism and certain assumptions regarding an exponential decay of the whole-body residence time. Thomas et al. [34] proposed using whole-body external counting as a predictor of blood concentration and clearance and eliminating blood samples from the dosimetry protocol. A camera-only approach, without blood sample measurements, was proposed by Van Nostrand et al. [15] whereby the whole-body retention at 48 h obtained from external counting could aid in the adjustment of the applicable empirical therapeutic doses previously derived by Sisson et al. [35]. Nichols et al. [2] determined that the calculation of MPA based on blood counts alone is comparable to MPA obtained from combined body counting and blood measurements, and therefore, blood counting alone is sufficient; this simplifies the dosimetry protocol. Kuker et al. proposed that an abbreviated dosimetry protocol of whole-body imaging and blood sample measurements up to 48 h is feasible in those patients with I-131 biological excretion greater than 50% at 48 h [36].

## 4. Methods and Materials

This is a retrospective analysis of data acquired at our Institution between 1996 and 2023, consisting of 83 adult patients (Table 1) who underwent I-131 dosimetry to calculate the MPA for I-131 RAIT planning. The Institutional Review Board of the University of Miami approved this study (IRB 20230984), and the requirement to obtain informed consent was waived. All data were handled in compliance with the Health Insurance Portability and Accountability Act (HIPAA) of 1996.

The method we used at the University of Miami/Jackson Memorial Hospital since the early 1990s is based on the bone marrow dose-limiting approach and involves blood drawing and gamma camera imaging over 5 or more consecutive days. The calculation of the MPA is performed using a customized implementation of MIRD formulism that ensures a 2 Gy upper threshold for blood-absorbed dose while also estimating the dose to remnants and lesions and considers an upper limit of 80 mCi of I-131 activity in the lungs at 48 h post-treatment.

Prior to I-131 administration, 3–4 mL of blood is drawn and counted in a well-counter for initial background determination. Subsequently, the patient is administered 2–3 mCi of a diagnostic I-131 dose in pill or liquid form. A source standard with a small amount of I-131 activity is also prepared to be included in the imaging field of view on the side of the patient. Whole-body imaging in the anterior and posterior views is performed simultaneously at 4, 24, 48, 72, and 96 h post-administration of the tracer dose of I-131, using a dual-headed gamma camera (ADAC-Philips Vertex Plus) with a 5/8 “NaI(Tl) crystal, high-energy collimators, and a 20% energy window. After image acquisition, the images are transferred to a nuclear medicine workstation for region of interest (ROI) analysis by outlining the body contour as well as the source standard in both the anterior and posterior views. Additional ROIs are drawn on the 48 h images over the chest, including the lungs. The generated counts from each ROI are decay-corrected to account for physical radioactive decay for the duration of the imaging study. Blood samples of 3–4 mL are drawn at each imaging point and within 30 min upon completion of the scan. At the end of the imaging study (typically at the 96 h mark), all the blood samples collected are pipetted appropriately and counted in a well-counter along with an I-131 calibration capsule and a 0.5 mL water sample for background. For the conjugate anterior and posterior views of the whole-body and the source standard, the geometric mean of the counts is calculated. The software calculates the MPA, which is not necessarily the prescribed treatment dose activity, and it might have to be adjusted for reasons related to the patient’s clinical condition, as discussed between the treating physicians.

## 5. Artificial Intelligence System

**Figure 1:**
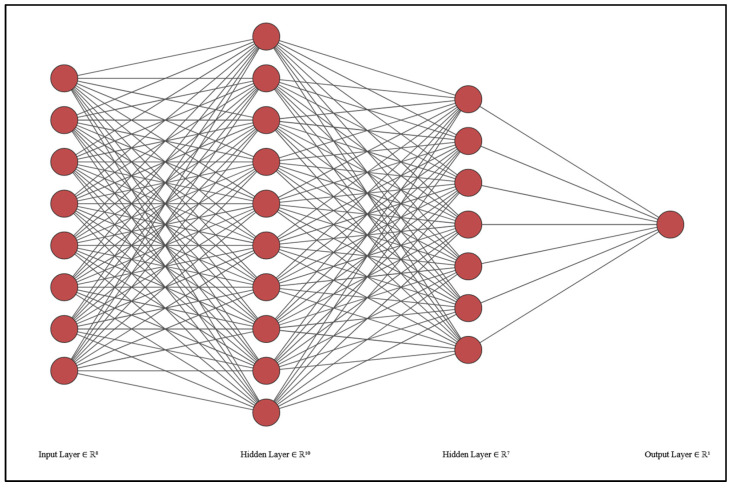
The AI model is designed with 8 input layers (counts and blood samples at 4 h, 24 h, and 48 h, as well as age and patient weight), two hidden layers, and an output layer for the result of the predicted MPA.

An AI system was developed with the aim of optimizing the current dosimetry protocol at our institution by limiting the number of patient visits for imaging to 4 h, 24 h, and 48 h only and eliminating the 72 h and 96 h. This is accomplished by using the imaging counts and blood sample measurements at 4 h, 24 h, and 48 h to predict the MPA as it would be obtained from the full set of measurements, including the 72 h and 96 h time points. Furthermore, we aim to use AI to explore possible inherent relationships between the different types of input data, such as camera counts and blood sample measurements, that were previously unknown. We employed a Python Keras neural network to perform the prediction of MPA. The network architecture consists of an input layer, two hidden layers with 10 and 7 nodes, respectively, and an output layer representing the calculated MPA (Figure 1). The input layer accepts eight features as inputs, including normalized anterior/posterior counts; corresponding blood sample counts at 4 h, 24 h, and 48 h; age; and patient weight. The first hidden layer consists of 10 nodes, and the second hidden layer consists of seven nodes. These layers are designed to capture complex relationships and patterns in the data. The activation function used for the hidden layers is ‘relu’ (Rectified Linear Unit), which introduces non-linearity and helps the network learn complex mappings between inputs and outputs. The output layer contains a single node representing the predicted MPA value. To train the neural network, we used the ‘adam’ optimizer and the ‘mean_squared_error’ loss function. The ‘adam’ optimizer is an extension of stochastic gradient descent that adapts the learning rate dynamically and has been shown to converge quickly and perform well on a wide range of problems. The ‘mean_squared_error’ loss function calculates the mean squared difference between the predicted and actual values, which is suitable for regression tasks. The training process involved iteratively updating the network weights based on the optimization algorithm. We used a training data set of 70 patients to train the model with consideration of a wide range of MIRD-calculated MPA values, from lower to higher amounts, to ensure the model is exposed to as many associations of the data parameters as possible. During training, the network learned to minimize the loss function by adjusting the weights to minimize the prediction error. To prevent overfitting, early stopping is implemented as a callback in the model. The training process is monitored for the validation loss, and if there is no improvement in the loss for 50 consecutive epochs, the training is stopped, and the model is restored to the weights that achieved the best validation loss. After training, we evaluated the performance of the trained neural network using a testing data set of 13 patients that was previously unused and unknown to the model. We computed the predictions for the test instances and compared them with the corresponding ground truth MIRD-based values. Statistical analysis was performed using Microsoft Excel to quantitate the accuracy of the values obtained using the AI system in comparison to the ones using MIRD with two tests: (a) a paired *t*-test at the 95% confidence interval (CI) and a Wilcoxon Signed-Rank test.

## 6. Results

**Figure 2:**
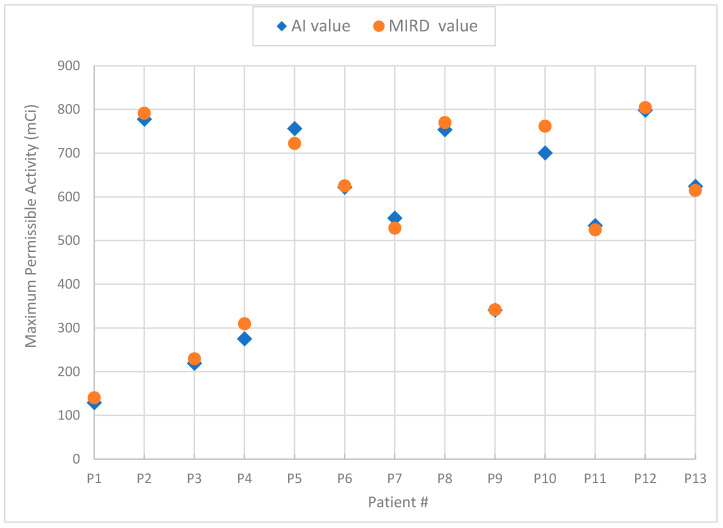
Graph of the MPA results generated by the AI system vs. MIRD for the validation data set.

Figure 2 is a graphical plot of the results showing the differences between the “actual” values as obtained with the conventional MIRD-based technique versus the AI-generated values produced by the DL system for the 13/83 patients that constituted the unknown set of testing data.

The paired *t*-test showed no significant difference between the MIRD values and the AI-generated results, with a value *p* = 0.351 at the 95% CI (range: −21.05 to 8.08 mCi). Due to the relatively small sample size, the Wilcoxon Signed-Rank test was also applied to examine the median percent differences between the two data sets, and no significant differences were found. Statistical analysis showed that the differences in the estimated MPA from the AI system compared to the MIRD-based approach, which was considered the ground truth, were less than ±10%, which is considered adequately acceptable. The comparison of the two methods, AI vs. MIRD, for the validation data set of 13 patients is shown in Table 2.

**Figure 3:**
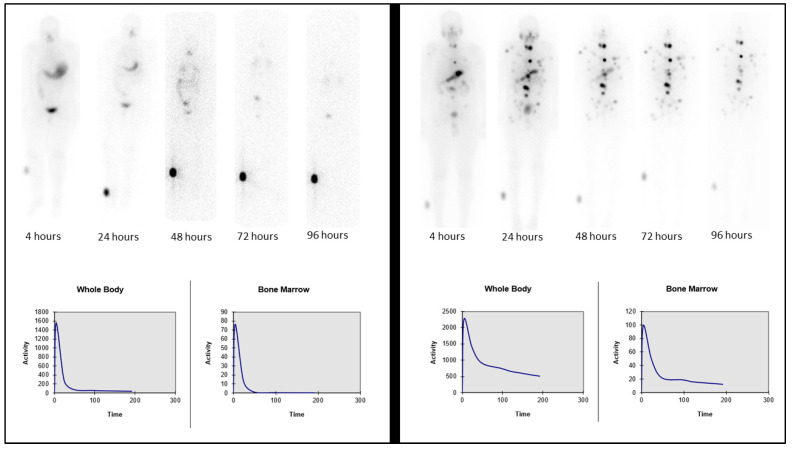
Two sample cases: P12, fast excretion and low residence time (**left**), and P3, slow excretion and longer residence time (**right**). Whole-body images (**top row**) and corresponding graphs for whole-body and bone marrow retention (**bottom row**).

Two case examples are shown in Figure 3 to demonstrate the performance of the AI system in predicting the MPA value for P12 with a low residence time (AI value of 798 vs. MIRD value of 804 mCi) and P3 with a high residence time (AI value of 219 vs. MIRD value of 230 mCi), effectively demonstrating a 1% and 5% difference, respectively.

## 7. Discussion

Dosimetry-guided RAIT is considered a safe and effective method for calculating the treatment dose that will deliver sufficient radiation to treat lesions while safeguarding against unwanted effects from excessive radiation, such as radiotoxicity to bone marrow and other tissues. Dosimetry protocols are generally considered complex and inconvenient for the patient, and thus great efforts have been made to simplify and streamline them.

Artificial Intelligence (AI), a discipline that, in its broadest definition, encompasses the development of systems that can exhibit human-like characteristics such as intelligence or behavior [37], has been increasingly impacting a wide range of applications. AI is poised to bring about a paradigm shift in all facets of society, with especially transformative changes to the medical field [38], including radiology and nuclear medicine [39,40]. With regard to nuclear medicine, AI-based algorithms show promise for implementation in many areas, such as image acquisition, reconstruction, post-processing, and segmentation, as well as diagnostics and prognostics [41]. Machine learning (ML) is a subdomain of AI involving techniques and processes that help a machine or system develop its own logic through self-learning [37]. Artificial neural networks (ANNs) are advanced ML algorithms that aim to mimic the biological structure of the brain in terms of layers of neurons [37], which are computational units that can process input data in complex ways using non-linear transformations to produce specific outputs [42]. Complex ANNs, typically involving multiple layers, can be used for the design of deep-learning (DL) systems. Such DL systems can be applied to complex problems requiring data analytics with the objective of optimizing techniques or methodologies. They can facilitate the design of systems that can be trained to calculate a particular output based on input data parameters without providing the conventional algorithmic process of how the data output was obtained in the first place.

In this investigation, we examined the feasibility of employing AI for optimizing the dosimetry protocol at our institution, by including imaging counts and blood sample measurements at the 4 h, 24 h, and 48 h time points and eliminating those at 72 h and 96 h. The AI system was trained on a data set of 70 cases from previously acquired patient data, and using design parameters that aimed to develop the necessary relationships and associations between the various data points and layers, it produced the predicted MPA for the RAI treatment as output. There was no statistically significant difference in the calculated MPA values between the AI model and the MIRD methodology. Following the MPA obtained by the MIRD method, the current workflow at our institution requires additional discussion between treating physicians to decide the final dose. This same process would apply to AI-obtained MPA values as an extra layer of caution in ensuring the final treatment dose is appropriate. The AI-based RAIT dosimetry approach offers several advantages, including the potential to reduce the number of patient visits and imaging sessions, streamline resource allocation, and enhance patient convenience without compromising dosimetric accuracy. The aim of this study was to provide a proof of concept of how an AI system could be developed and implemented to optimize and simplify existing dosimetric protocols.

The developed AI system has some limitations that are worth noting. The system was trained using a data set of 70 cases and tested with a data set of 13 previously unknown cases. Even though there are no systematic differences between the two data sets other than their number of samples, it would be expected that the accuracy of the developed AI system could be further improved with a larger training data set. The predictive reliability and accuracy of such AI models are generally enhanced when they are trained and validated with diverse and comprehensive data sets [40]. The generalizability of the AI system, defined as the system’s ability to properly work with previously unseen data such as from other scanners or institutions or acquired using different protocols [42], should also be tested and evaluated.

Future work for the AI system includes expansion using a larger cohort for both testing and validation and also the augmentation of input parameters with additional information, such as the creatinine levels of the patient. Additionally, other optimization possibilities will be explored, such as the intricate role of blood sample measurements in the image counts and whether they could be eliminated to further streamline the protocol.

## 8. Conclusions

In conclusion, nuclear medicine plays a key role in thyroid cancer management using molecular imaging and radiopharmaceutical therapies that are successfully applied for DTC as a standard of care. Radioiodine dosimetry for RAIT offers more precise and personalized planning for effective treatment in patients with DTC. The current study demonstrated as a proof of concept that it is feasible to incorporate AI methods to optimize and simplify current dosimetry protocols, thus making them more widely clinically accepted while also improving patient experiences. Future work is warranted to assert the generalized applicability and validation of the proposed AI method.

## Figures and Tables

**Table 1:** Characteristics of the RAIT patient cohort.

No. Sex, Age, y	All	Male	Female
Patients (*n*)	83	30	53
Mean Age (y)	48.2	45.9	48.8
Mean Weight (lbs)	161.1	181.4	149.6
Average Calculated Maximum Permissible Activity (mCi)	456 (range 117–1080)		

**Table 2:** Comparison of MIRD-based vs. AI-based MPA values for the 13 test patients.

Patient #	P1	P2	P3	P4	P5	P6	P7	P8	P9	P10	P11	P12	P13
MIRD (mCi)	140	791	230	310	722	625	529	770	342	762	525	804	615
AI (mCi)	129	778	219	275	756	622	551	754	341	701	534	798	624
Difference (mCi)	−11	−14	−10	−35	34	−3	22	−16	−1	−61	9	−6	9

## Data Availability

Data are obtained within the article.
